# A Comparison of the Spatial Linear Model to Nearest Neighbor (k-NN) Methods for Forestry Applications

**DOI:** 10.1371/journal.pone.0059129

**Published:** 2013-03-19

**Authors:** Jay M. Ver Hoef, Hailemariam Temesgen

**Affiliations:** 1 Alaska Fisheries Science Center, NOAA Fisheries, Seattle, Washington, United States of America; 2 Department of Forest Engineering, Resources and Management, College of Forestry, Oregon State University, Corvallis, Oregon, United States of America; Universitat Rovira i Virgili, Spain

## Abstract

Forest surveys provide critical information for many diverse interests. Data are often collected from samples, and from these samples, maps of resources and estimates of aerial totals or averages are required. In this paper, two approaches for mapping and estimating totals; the spatial linear model (SLM) and k-NN (k-Nearest Neighbor) are compared, theoretically, through simulations, and as applied to real forestry data. While both methods have desirable properties, a review shows that the SLM has prediction optimality properties, and can be quite robust. Simulations of artificial populations and resamplings of real forestry data show that the SLM has smaller empirical root-mean-squared prediction errors (RMSPE) for a wide variety of data types, with generally less bias and better interval coverage than k-NN. These patterns held for both point predictions and for population totals or averages, with the SLM reducing RMSPE from 9% to 67% over some popular k-NN methods, with SLM also more robust to spatially imbalanced sampling. Estimating prediction standard errors remains a problem for k-NN predictors, despite recent attempts using model-based methods. Our conclusions are that the SLM should generally be used rather than k-NN if the goal is accurate mapping or estimation of population totals or averages.

## Introduction

Forest surveys provide critical information for many interests: quantifying carbon sequestration, making sound management decisions, designing processing plants, guiding decisions among conflicting land uses, and quantifying wildlife habitats, just to name a few. To meet national and international negotiations and reporting requirements, forest management plans require local inventory data on vegetation, site productivity, biomass, carbon and other resources. The data must be intensive enough to include structural variables relevant to biomass and carbon projections and extensive enough to cover hundreds to thousands of acres, but can not be too expensive to collect. Thus, data are often collected from samples. From these samples, maps of resources and estimates of aerial totals or averages are required. One approach to mapping and estimating totals of biomass and productivity data is a spatial linear model (SLM), which includes ordinary kriging and universal kriging. This approach was initially developed for a similar goal: to predict geographic values or totals for mining resources. However, another approach, k-NN (k-Nearest Neighbor), has been recently developed and has gained widespread use. The overall goal of this paper is to compare SLM to k-NN theoretically, through simulations, and as applied to real forestry data.

The k-NN method finds observed samples that are “close” to an unobserved location based on covariates, and then either imputes the “closest” one directly as a prediction (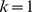), or forms a weighted average as a prediction (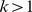). Widespread availability of remotely-sensed data as covariates allows extending ground information to large areas using k-NN. One of the reasons k-NN is popular is that, when 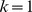, predictions are within the bounds of biological reality because they were observed in the samples [1]–[3]. Also, the logical relationships among response variables will be maintained, so k-NN is a multivariate method that retains the variable relationships seen in the data, particularly when 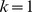 [1], [4]–[6]. When variables are predicted separately, the dependence structure among the response variables is generally lost [7]. The multivariate aspect of the k-NN may be necessary for inventory applications where information on multiple stand attributes is required for stand management decisions or further modeling [5], [8]. Because k-NN methods reuse existing samples, they are distribution-free [2], [9], [10]. Non-parametric k-NN imputation methods may provide better matches to listings of tree species for complex stands with multiple species and a wide variety of tree sizes, which tend to have multi-modal distributions [8]. Non-parametric methods were found to effectively describe local conditions and variability [11], [12]. One way to make k-NN local is to select a combination of neighbors from the neighborhood where the average of the covariates is closest to the target record covariates [12], [13]. Localization can also be achieved by using spatial coordinates as covariates or by restricting the selection of neighbors to a circular area around the target unit [14]. The yaImpute R package [15] facilitated the comparison of different k-NN approaches and its wide use.

There are some recognized problems with k-NN. It tends to be highly biased at the edge of the data cloud because prediction sites will likely be paired with a more central sample value due to the asymmetric neighborhood [16], [17]. Extremely small values and extremely high values will be over- and underestimated, respectively, if the sample data do not cover the whole range of variability [18], [19]. Bias can also be a problem in the interior of the data cloud if the covariates are non-uniformly distributed [20]. In k-NN methods, the match is found using the covariates that are available for every site. As the number of samples increases, there will be a higher chance of getting an exact match in the covariates. However, this only guarantees an exact match for the response variables if there is perfect (or very strong) correlations with the covariates. As sample size increases, there is no guarantee that the mean of the response variables will approach the true mean and hence k-NN methods are not statistically consistent [3]. The k-NN methods lack a good measure of uncertainty, and often the global root-mean squared error from cross-validation is used for point-wise standard errors [20]. However, that global root-mean squared error may not be a good measure of accuracy when response variables have heteroscedastic variances around the covariates; it was recommended that graphical tools be used to evaluate issues of bias, homoscedasticity, influential observations, outliers, and extrapolations [21]. An approach that uses a model of the covariates space variogram to quantify prediction uncertainty was proposed, but is computationally demanding [22]. Relevant accuracy statistics for assessing the quality of predictions of categorical variables are still lacking [7]. However, there are recent proposals for a variance estimator that incorporates spatial correlation [23], model-based estimators of the uncertainty [24], and design-based approaches to derive the statistical properties of the k-NN predictions [17]. If 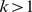, then k-NN loses many of its purported benefits as some estimates may not be within the realm of real values [3]. If there are several response variables or “rare” polygons (stands), a good match will be very difficult to find [3].

Geostatistical methods were also devised for prediction, both for single sites or block averages. For a history, see [25]. Combining the notion of classical geostatistics with a linear model (e.g., regression) yields the spatial linear model (SLM) (called universal kriging in the geostatistical literature). In comparison to k-NN, SLM predictions were designed to minimize the root-mean-squared error. If the data-generating process is multivariate normal, then the SLM predictor is equivalent to the conditional expectation, which is optimal [26](pgs. 108–110). Moreover, if the generating mechanism is not multivariate normal, the SLM predictions are still optimal among the class of linear predictors, called best linear unbiased prediction (BLUP) [26](pgs. 151–155). The BLUP was extended to finite populations of spatial data [27]–[29]. Although distribution-free, BLUP requires specification of a covariance model; however predictions are generally robust to mis-specification of the covariance model [30], [31]. Classically, geostatistical methods estimated the covariance model by binning data into distance classes and using a least-squares fit [32]; however, restricted maximum likelihood (REML) estimation [33], [34] removes the arbitrary nature of binning and fitting and is an unbiased estimating equation approach to the SLM [35], [36]. Finally, SLM predictors are linear, creating weighted averages of data, and we can appeal to a spatially correlated version of the central limit theorem that predictors will be asymptotically normally distributed, e.g. [37], allowing for inference based on a standard normal distribution (e.g., for prediction intervals). More detailed and mathematically-oriented arguments on the stability of the geostatistical method can be found in [38] and [26](pgs. 289–299). There are many extensions of the SLM when predictions cannot be assumed approximately normally-distributed, e.g. [39].

The SLM is not without some recognized problems. Most of these are related to the problem of estimating the spatial covariance function. In a sense, the data are used twice; once to estimate the covariance parameters and secondly used for BLUP. This has been termed empirical best linear unbiased prediction (EBLUP) [40]. Forming multivariable models requires estimating many covariance parameters, and any gain in precision is often minimal [41], with gains often 10% or less [42]. However, see [43], [44] for forestry applications. Some claims against the SLM are in error. Some forestry data are obtained from polygons that do not have a unique position in space and are irregular lattice data (aggregates of spatial data). Some authors, e.g. [6], indicate that the SLM is not appropriate for these data, but that is not correct. Polygon centroids can be used to compute distance, as shown with the real forestry data below. We note, however, that this may be a problem for very irregularly shaped polygons.

It turns out that predictors for both SLM and k-NN are linear, and the above review indicates that SLM should be optimal. However, much of that theory is based on an assumed spatial stochastic model, does not take into account the estimation of the covariance parameters, nor does it say how much better SLM might be. k-NN has fewer assumptions, and perhaps it is easier to implement and faster to compute, and a small loss in efficiency is compensated by ease of use and computational speed (see [45] and references therein). We build on a few previous comparisons [46], [47], where they compare only for point predictions, they do not assess the validity of prediction standard errors, and they use variogram estimators on residuals, which is known to be biased for covariance parameters [48](pgs. 257–258). One problem in making the comparison is that k-NN is an algorithm that makes no assumption about how spatial data were created; it only assumes a fixed surface. The SLM, on the other hand, is based on the idea that data are a realization of a spatial stochastic model. By conditioning on realizations under a SLM, and hence adopting a fixed surface perspective, k-NN and SLM can be evaluated in a common framework. Foresters and forest managers are interested in the global performance of predictive or imputation methods across a management region, among management regions, and across time. Supporting that practice, the comparison of prediction or imputation methods using global computations of average bias and RMSPE (described in Section “Performance Measures”) is deeply rooted in the forestry literature [3], [10], [14], [21].

Our goal is to compare k-NN to the SLM using simulations of artificial populations and resampling real forestry data. In the Methods section, we make explicit the prediction goals and k-NN and SLM methods and models, and we describe the simulation methods and real forestry data. The outcomes of using k-NN and SLM on these data are presented in the Results section. We discuss the results and offer some conclusions in the Discussion section.

## Methods

We will use the following notation for spatial data. Let the population of response values be partitioned into those that are observed 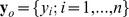 and those that are unobserved 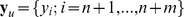, and 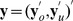. Let the index set for the observed data be 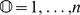 and for the unobserved data be 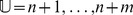. We consider two main goals: 1) point prediction of 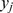 for 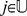, and 2) block prediction of the total or average 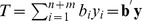, where 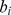 are the weights that define the block objective; e.g., if 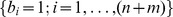 then 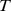 is a population total, and if 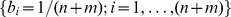 then 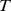 is a population average. Note that prediction goals for small areas can also be defined using zeros as weights in 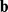, but we do not pursue that here. For all response values, there are covariates contained in a design matrix 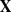 which has 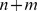 rows and 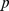 columns, where the first 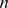 rows, 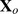, correspond to 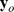 and the next 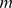 rows, 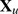, correspond to 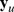. The spatial coordinates are contained in the matrix 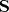 which has 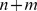 rows and 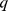 columns, where the first 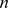 rows correspond to 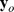 and the next 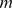 rows correspond to 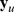. In 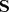, 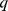 is generally two. For example, the first column could be longitude and the second column latitude, or some planar transformation of them. A coordinate such as height might also be included, but we do not consider it here.

To meet our two objective above, we define the linear predictor,
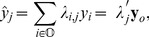
(1)where 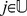. A linear block predictor is



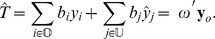
(2)As will be seen, both k-NN and SLM are linear predictors. Note that [23] and [24] attempt model-based estimators for a version (2) that includes predicted values of 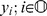 rather than simply summing the observed values. The variance of such predictors lack any notion of a finite population that shrinks the variance as the sampling fraction increases; i.e., we want a variance estimator such that 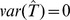 if the whole population is observed, so we do not pursue their formulation any further.

Both the SLM and k-NN use distance in various ways so a general definition is given here. Let 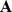 be a matrix with coordinates in the columns and the 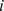
th row denoted as 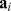. A general distance formula between the 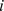
th and 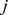
th rows of 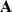 is,
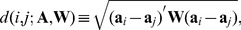
(3)where 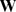 is a weighting matrix.

### Review of k-NN

Let 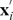 and 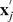 be the 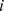
th and 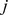
th rows of 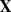, respectively. Then a “variable-space distance” between the 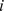
th and 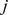
th sites can be computed as 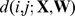. Several types of distances are possible. For example, if 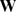 is the identity matrix, this is raw distance; if 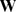 is diagonal with the inverse of the empirical variance for each of the columns in 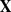 as the diagonal elements, then this is normalized distance; if 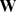 is the inverse of the empirical covariance matrix among the columns in 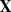, then this is Mahalanobis distance; and if 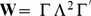, where 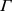 is the matrix of canonical vectors from canonical correlation analysis between 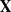 and 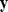 and 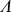 is the canonical correlation matrix, then this is the “most similar neighbor” (MSN) distance [1]. All of these and others have been implemented in the yaImpute package [15] in R [49]. The k-NN method chooses weights 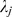 based on a distance matrix. Let 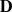 be a distance matrix with 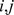
th element 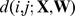, which can be partitioned as
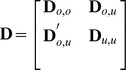



Let 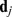 be the 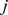
th column of 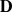, 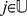, contained in 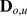; i.e., 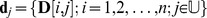. If 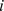 is the index for 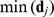, then for a first-order nearest neighbor, 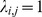 in (1) and all other 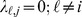. This essentially assigns the value of 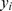 to 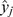 for the 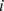
th site that is closest to the 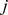
th site in variable-space. Let 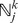 be the index set of the 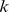 nearest sites (smallest values) in 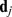. Then 
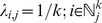
 takes the average of 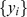 from the 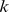 nearest neighbors in variable-space. Another option is to weight inversely proportional to distance, where 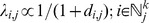 where 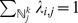 and 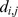 is the 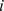
th element in 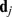.

Cross-validation is the method most often used to compute prediction standard errors [20]. Cross-validation makes predictions for sites that already have values, where each sample is removed one at a time, and the rest of the sample is used to predict the one that was removed. The idea is to use in-sample averaged squared errors between the predicted and observed values to serve as a global estimator of squared errors when out-of-sample. Let the k-NN prediction standard error be estimated as,
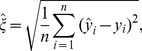
(4)where 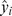 is the cross-validation prediction of 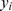 for 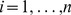 sample values. Assuming prediction errors are normally-distributed, 90% prediction intervals are formed as 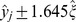 for 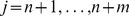 out-of-sample values. Note that these are constant for all 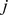; we examine a spatially explicit model-based approach [23] in Section “A geostatistical approach for estimating the variance of k-NN predictors.”

For the standard error of estimating a total, we borrow from the idea of classical sampling theory, e.g. [50], where 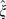 replaces the standard error,
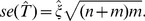
(5)


### Review of SLM

Assume only the linear model
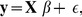
(6)where 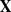 is a matrix of fixed covariates, 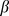 is a vector of parameters, and 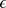 is a random vector with 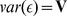 for some unknown spatial multivariate distribution. Note the contrast to k-NN; (6) is a spatial stochastic model that allows optimization with respect to bias and squared error, which we now review. Let 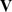 be partitioned as
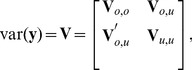
(7)and let 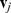 be the 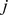
th column of 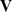, 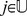, contained in 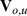; i.e., 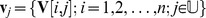. Consider squared-error loss, and the linear predictor 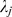 in (1), where 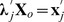 and 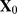 contains at least a column of ones. The predictor that minimizes squared-error loss, known as the best linear unbiased predictor (BLUP), 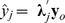, has
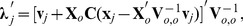
(8)where 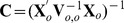, with prediction variance of



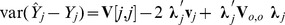
(9)
[26] (pgs. 151–155). Notice that 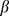 is unknown; the only assumption is the linear model and a known spatial covariance matrix.

Assume the same linear model in (6), except this time the linear predictor is (2). Let 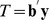, where 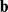 is a vector of all ones, and 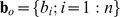 and 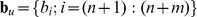. For a finite population the BLUP, 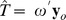, has
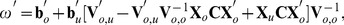
(10)with prediction variance of
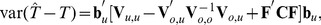
(11)where 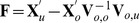 [27]–[29]. The finite population correction factor is not obvious in (11). However, as 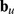 gets shorter in length, (11) goes to zero. If 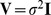, then (11) simplifies to 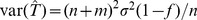 where 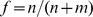 is the sampling fraction; this is the classical formula in simple random sampling without replacement for finite populations, e.g. [50](pg. 16).

For equations (9) and (11) 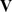 is unknown and must be estimated. In spatial models, 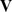 is modeled through spatial information; in geostatistics this is spatial distance. Consider the exponential autocovariance model,
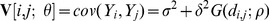
(12)where 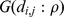 is a general autocorrelation function, 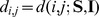 as defined in (3), 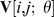 is the 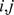
th element of 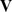, 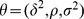 with 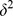 as the partial sill, 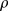 as the range parameter, and 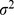 as the nugget effect (which may absorb spatial autocorrelation at very fine scales within minimum sampling distances). We will fit models using 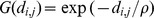; for many other models see [51](pgs. 80–96). The larger 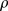, the more autocorrelation between sites for a given distance. The parameters 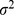 and 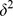 are variance components, with 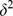 controlling the autocorrelated component and 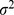 controlling the uncorrelated component. For all models in this article, we use (12), relying on the fact that inferences are generally robust to mis-specification of the model. We estimate the covariance parameters using restricted maximum likelihood (REML) [33], [34],
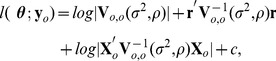
(13)where 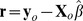, 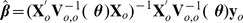, the dependence of 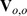 on 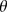 is denoted as 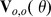, and 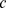 is a constant that does not depend on 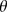. Equation (13) is an unbiased estimating equation [35], [36] and minimizing it for 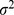 and 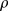 provides their REML estimates. Using the estimated covariance parameters from REML in equations (8–11) provides the EBLUP predictors and standard errors.

The significance of using (13) is three-fold: 1) normality is not required to use (13) because it is an unbiased estimating equation, 2) there is no need to de-trend because estimation of 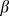 is essentially embedded in (13), and 3) there is no need to compute empirical variograms by binning residuals. Residuals from de-trending are biased [48](pgs. 257–258) and binning is arbitrary. Thus, (13) provides an automatic way to estimate a spatial covariance matrix in very general conditions.

### A Geostatistical Approach for Estimating the Variance of k-NN Predictors

An iterated variogram estimator for the variance of a k-NN predictor has been proposed [23]. Suppose that we start with iteration 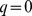 and (4) as an estimator for a constant prediction standard error, 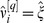. Form standardized residuals as
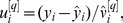
(14)where 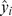 is the in-sample cross-validation prediction value using some k-NN method. Then compute an empirical semivariogram,
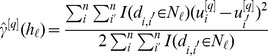
(15)where 
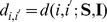
, the 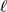
th distance class is 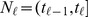, where 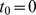, 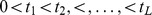, and 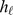 is some function of 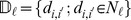; e.g., 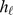 might be mean of all distances in 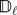, the median of 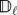, or the midpoint 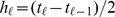. A semivariogram, such as the equivalent to (12),
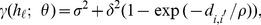
(16)is fit to (15), typically by minimizing a weighted-least-squares criteria; e.g. [32],



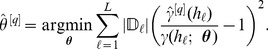
(17)Let the spatial autocorrelation be 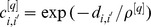. Then a local estimator of variance is
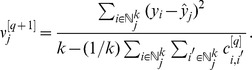



Now go back to (14) with updated 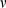
’s and iterate until convergence. For a convergence criteria, we used
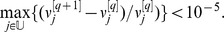



After convergence, a local estimator of prediction variance is
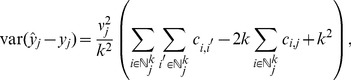
(18)where the iteration superscript 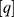 is suppressed after convergence. The prediction variance estimator (18) can be seen as an attempt to form a local (in covariate space) version of (9) without having to estimate mean effects due to “nearness” in covariate space. Note that this estimator will not work for 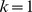, and it is only sensible when the mean of nearest neighbors is computed, as compared to the distance-weighted version. However, the above method could be adopted for distance-weighted. The estimator (18) will be examined using simulations. For the simulations, we used 10 equal-interval variogram distance-bins (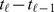 was equal for all 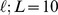) between 0 and the maximum distance in the data set. Equation (17) was minimized using the optim() function in R [49] with the Nelder-Mead simplex method [52], obtaining a starting value from a 10×10×10 search grid for the parameters of (16). A maximum of 30 iterations was allowed.

A more formal geostatistical analysis of the k-NN predictors is as follows. The k-NN predictor can be written as (1). Under the SLM, the root-mean-squared prediction error (RMSPE) is
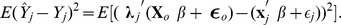



After taking expectations,
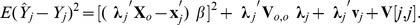



Note that k-NN methods make the sensible constraint that 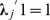 when using the mean of the nearest neighbors or distance-weighting. If 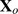 is a single column of ones, then the bias term 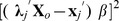 above disappears. However, this is not true in general. In contrast, under the BLUP, the bias term disappears due to further constraints on 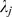 that guarantee unbiasedness for any 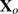 and 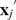. Hence, the RMSPE for k-NN will be greater than BLUP for two reasons: it is not optimized for minimizing the error variance 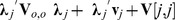 and there is a bias-squared component.

An estimate of the RMSPE of the k-NN predictor under the SLM model is obtained by replacing 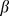 with 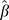. Note that
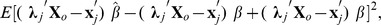
which equals



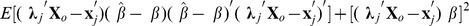
because 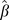 is unbiased for 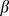. Note that 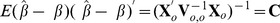, so
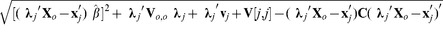
(19)is an estimator of the RMSPE of a k-NN predictor using parameters estimated under a SLM; i.e., covariance parameters can be estimated using (13) and 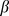 can be estimated using generalized least squares, 
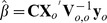
. We will analyze some k-NN estimators using (19) in simulations below.

### Simulation of Artificial Data

We created spatially-patterned and cross-correlated 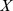-variables. All data sets were repeatedly simulated on a 20×20 regular grid evenly spaced between −1 and 1 on both coordinate axes and eight covariates: 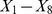, described next. Start with
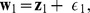
where 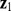 is a 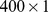 vector of values (on the 20×20 regular grid) containing zero-mean spatially-autocorrelated random variables from some geostatistical model with partial sill 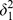 and range parameter 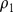, and 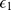 is a 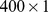 vector containing zero-mean independent random variables with variance 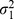. We also let 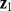 be independent of 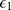. Next, we set up an autoregressive-like recursion, where



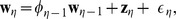
where 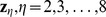 contains zero-mean spatially-autocorrelated random variables from some geostatistical model with partial sill 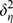 and range parameter 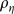, and 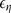 contains zero-mean independent random variables with variance 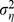, where again 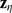 is independent of 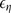. Note that 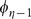 is a parameter that creates cross-correlation between variables by regressing 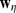 on 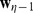. This set-up ensures cross-correlation among the 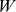-variables and spatial autocorrelation within each 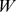-variable. Now let



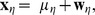
where all the elements of 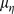 are constant, equal to 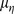. Finally, create the response variable as,
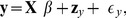
(20)where 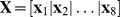, 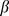 is a vector of parameters, and 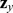 contains zero-mean spatially-autocorrelated random variables from some geostatistical model with partial sill 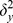 and range parameter 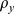, and 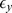 contains zero-mean independent random variables with variance 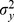, where again 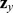 is independent of 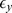.

For simulations, we let all 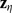 and 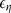 be normally distributed. For autocorrelation of 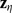, we used the spherical model,
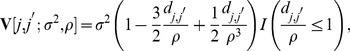
(21)where 
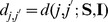


We simulated three types of data using these models. In all cases 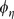 = (NA, 0.5, 0.5, 0.5, 0, 0.5, 0.5, 0.5). Note that for each simulated data set the covariates 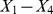 were cross-correlated through 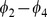, but 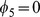 broke any further cross-correlation to the group 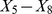, and then 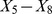 as a group were cross-correlated.

1. The first simulated method had 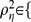
0.25, 0.5, 0.75, 1, 1.25, 1.5, 1.75, 2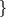, 
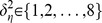
, 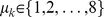 and 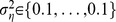, 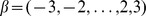, 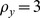, 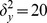, and 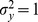.
2. For the second simulation method, let 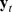 denote the simulated data, with 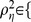
0.5, 1, 1.5, 2, 2.5, 3, 3.5, 4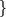, 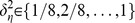, 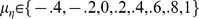 and 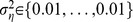, 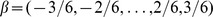, 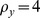, 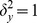, and 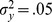. Then, 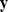 was simulated from Poi(exp(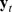)) where Poi() is a Poisson distribution. Note that 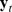 wandered well below zero over significant spatial patches creating zero-inflated count data. Variances and regression coefficients are smaller here to keep numbers reasonable when exponentiating.
3. For the third simulation method, let 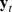 denote the simulated data, with all of the same parameters as for the second simulation. Then, 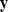 was simulated as 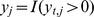, creating binary data of zeros and ones.

Note that the group 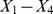 had smaller ranges and variances than group 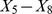. The response variable was related to both groups through 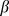, but the coefficients were zero for 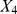 and 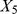. We made predictions for both k-NN and SLM methods using only 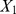, 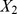, 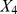, 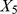, 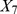, 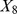. Thus, several model mis-specifications were made: inclusion of covariates with no effect (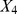 and 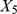), exclusion of covariates with real effects (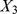 and 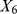), and in the case of SLM, generating data from a spherical autocovariance model but fitting the data with an exponential autocovariance model.

For each of the three simulation methods listed above, 2000 data sets were simulated (with 400 simulated values per data set). For each simulation 100 locations were sampled randomly, and from each sample the other 300 were predicted, along with the overall total.

### Forest Data

Data for this study were obtained from the Forest Inventory and Analysis (FIA) databases for Oregon. The FIA databases are part of the national inventory of forests for the United States [53], [54]. The Food Security Act of 1985 protects the confidentiality of the Forest Inventory and Analysis (FIA) plot locations, the integrity of the FIA sample, and the privacy of landowners who allow FIA field crews on their land. For these reasons, the actual plot locations are not available publicly. Persons needing exact plot locations to examine or reproduce our results should contact the Pacific Northwest Forest Inventory and Analysis Program at http://www.fs.fed.us/pnw/fia/. Alternatively, FIA produces and maintains a set of public databases with perturbed plot coordinates that can be downloaded and used by anyone. The website for accessing these data is http://www.fia.fs.fed.us/tools-data/.

A tessellation of hexagons, each approximately 2400 hectares in size, is superimposed across the nation, with one field plot randomly located within each hexagon. Approximately the same number of plots is measured each year, each plot has the same probability of selection, and in the western U.S. plots are remeasured every ten years. Each field plot is composed of four subplots. Forested areas that are distinguished by structure, management history, or forest type are mapped as unique polygons (also called condition-classes) on the plot and correspond to stands of at least 0.4047 hectare in size. For our study area there were 1886 forested FIA plots measured between 2001 and 2006. Biomass (DRYBIOT), maximum potential mean annual increment (PMAI), elevation, and primary species identifier (i.e., Douglas-fir (*Pseudotsuga menziesii*), ponderosa pine (*Pinus ponderosa*) or western hemlock (*Tsuga heterophylla*)) were obtained from the FIA annual database.

**Figure 1:**
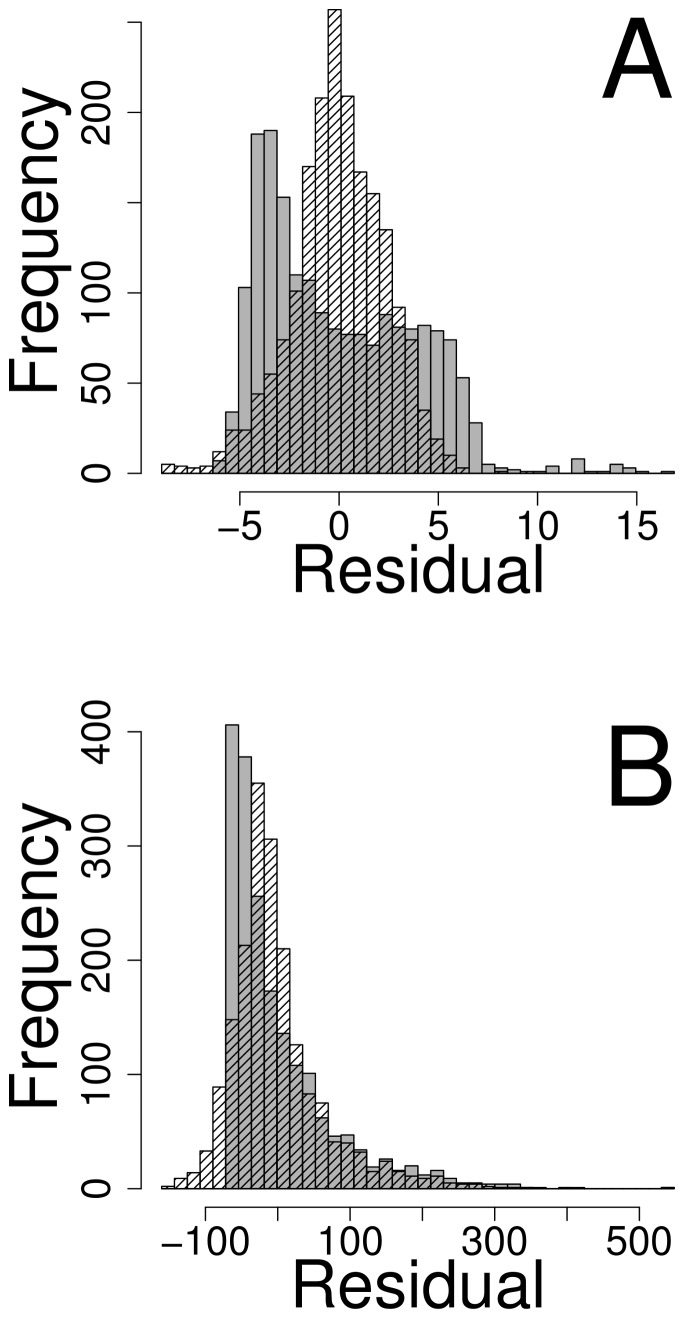
Histograms of A. PMAI, B. DRYBIOT. The gray-shaded histograms are based on the original centered data, and the cross-hatched histogram is based on the residuals after fitting a multiple regression model with main effects for all covariates.

For climate data, we used monthly temperature and precipitation normal data for the period 1971–2000 produced by the Parameter-elevation Regressions on Independent Slopes Model (PRISM), which provided an 800 m grid that produced differences between measured plot elevation and overlaid PRISM grid elevation up to 350 mm in the mountainous areas of Oregon. To account for changes in climate due to these elevation differences we utilized a process similar to [55] where we created a scale-free interpolation process using a 90 m digital elevation model and PRISM temperature and elevation gradients of the larger 800 m grid. The result is a 90 m monthly climate grid. Like [55] we used this procedure for extracting temperature (T) only and used a simple distance weighting method for precipitation (P). Climate Moisture Index (CMI), a measure of precipitation in excess of evapotranspiration, was used to quantify moisture availability. The raw values of PMAI and DRYBIOT, along with residuals after fitting a linear model, are shown in Figure 1; covariates (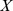-variables) in the linear model were temperature, precipitation, Climate Moisture Index (CMI), WH (an indicator variable for shade tolerance based on Western Hemlock trees) and elevation.

**Figure 2:**
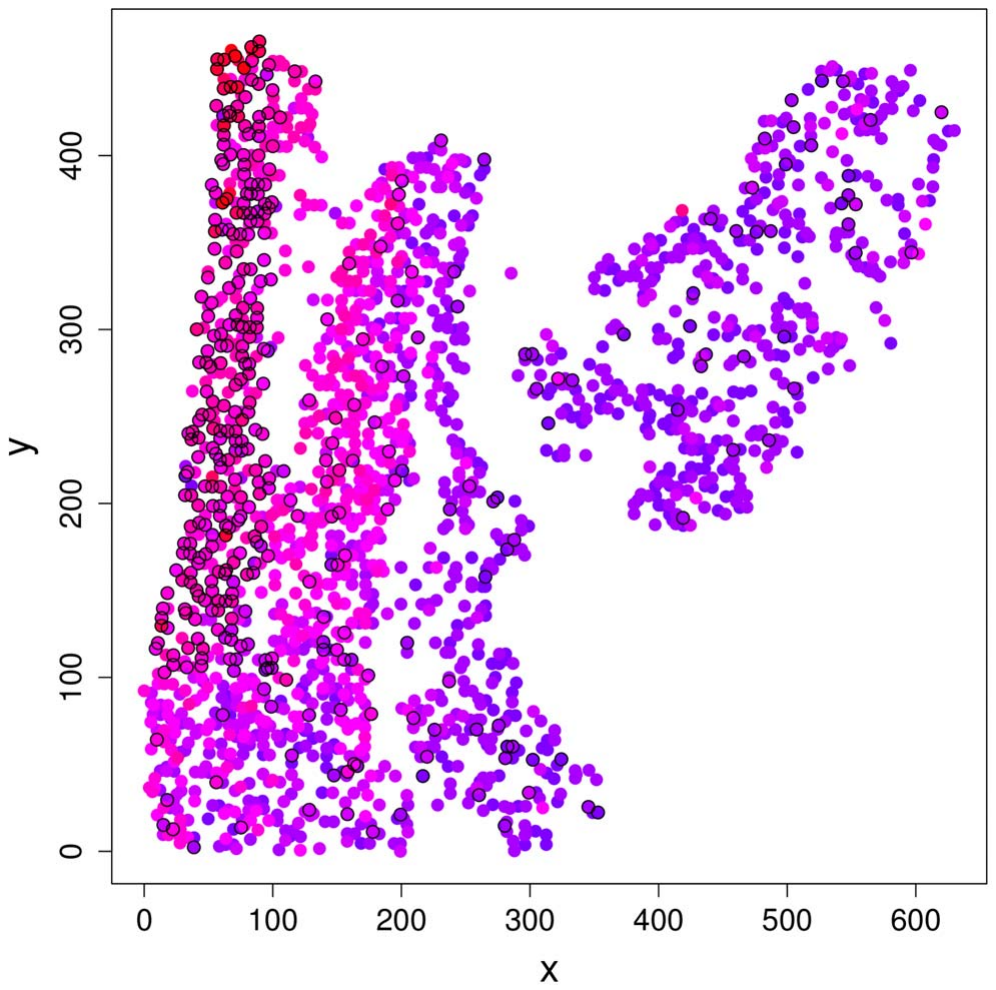
Spatial locations of PMAI variable. The redder shades indicate higher values, and the bluer shades indicate lower values. One draw from the unbalanced spatial sample is shown with black circles around the sampled locations.

For real forest data, we used a census of 1886 known FIA values for PMAI and DRYBIOT. These were subsampled in three different experiments. For each resampling experiment listed below, the data sets were subsampled 500 times. For each subsample, a sample of 386 values was chosen randomly without replacement, and from each sample the remaining 1500 locations were predicted, along with an estimate of the total for all 1886 values. The covariates used were temperature, precipitation, CMI, WH, and elevation. The 500 resamplings were performed for each of the following:

1. PMAI: this variable reflects the maximum potential forest productivity at a particular site. It indicates the average annual productivity of wood volume (m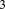/ha/year) that would be realized over time.
2. DRYBIOT: this variable is the total above-ground oven-dry biomass of live trees larger than 2.5 cm in diameter.
3. PMAI with unbalanced spatial sampling. Here, we preferentially sampled geographically, as often happens in real applications due to access or other issues. We divided up the study area into four parts, using 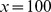 and 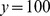 as the dividing lines. We created samples by sampling 200 values without replacement for 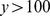 and 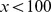, and then randomly sampling 186 values without replacement from the rest of area. One such sample is shown in Figure 2. From each unbalanced spatial sample, the remaining 1500 locations were predicted.

### Performance Measures

Over the six preceding simulation/resampling experiments, the SLM is compared to k-NN using three measures of predictive performance, both for individual site predictions, and totals over all sites. Note that these are simple summaries of predictive performance, and make no assumptions about how the data were generated; they are global summary statistics to help evaluate and compare methods. Let 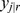 be the true, known value for the 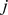
th location and the 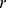
th simulation or resampling. Note that if we use 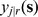 to denote the spatial location 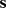 associated with 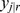, then 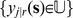 will vary over 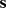 for each simulation/resampling because observed samples were randomized each time. We indicate this dependence on 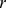 as 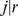. A prediction of 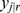, using (1), is denoted 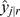 for each 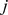 and 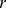. For each 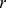, there is a true total 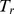, and an estimate, using (2), denoted 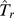 for the 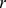
th simulation/resampling. The three performance measures are:

- RMSPE: the root-mean-squared prediction error measures how close the estimates are to the true values.

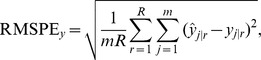
for point-wise predictions, where 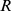 = number simulations (1500) of artificial data or resamplings (500) of real forestry data for point prediction, and 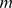 = number of point predictions (300 for artificial data and 1500 for real forestry data) per replication 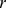, and

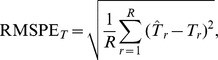
for total prediction. A smaller value of RMSPE indicates predictors are closer to true values.
- SRB: signed relative bias. Absolute bias is meaningless, so it is expressed as a fraction of the variability. It is well-known that MSPE = bias
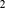
+variance, and we use MSPE as RMSPE squared from above. The sign of bias is also informative, so signed relative bias as a fraction of variability is computed as,

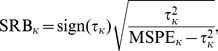
where

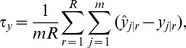
for point-wise predictions, and

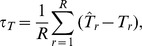
for total predictions, sign(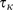) is the sign (positive or negative) of 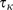, and 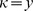 for a point-wise performance measure or 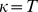 for a total performance measure. A smaller absolute value of SRB has smaller bias, and a negative sign indicates under-prediction and a positive sign indicates over-prediction.
- PIC90∶90% prediction interval coverage, measures how well uncertainty is being estimated. For many predicted values, or over many simulations, a prediction interval should cover the true value with the claimed proportion. For point-wise predictions, the empirical prediction interval coverage was computed as,

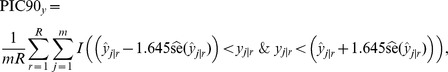
where 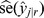 is the estimated standard error of 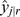, taken from (4) for k-NN methods, and from the square root of (9) for the SLM (EBLUP), with covariance parameters estimated from (13). PIC90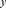 should be near 0.90 if prediction intervals are properly estimated. It is also possible to compute PIC95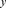 by replacing 1.645 with 1.96 in the formula above, and PIC95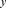 should be near 0.95. For total predictions,

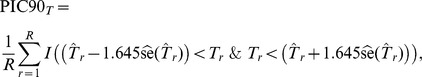
where 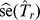 is the estimated standard error of 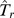, taken from (5) for k-NN methods, and from the square root of (11) for the SLM (EBLUP), with covariance parameters estimated from (13).

### Prediction Methods

Seven prediction methods were examined; five different k-NN methods, multiple regression (a special case of a SLM that assumes independence), and a SLM:

- MAH1: k-NN that uses Mahalanobis distance with 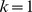.
- MAH5: k-NN that uses Mahalanobis distance with 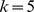.
- MSN1: k-NN that uses most significant neighbor (MSN) with 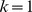.
- MSN5: k-NN that uses MSN with 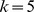.
- bstNN: k-NN that uses both Mahalanobis distance and MSN, and tries 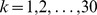, and then chooses the distance matrix and 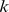 with the smallest cross-validation RMSPE from the observed data.
- SLM: a spatial linear model using the same covariates as all k-NN methods as main effects only, with an exponential autocovariance model estimated by REML, and using prediction and variance equations as described in the Review of SLM section.
- LM: multiple regression like SLM but assuming all random errors are independent.

## Results

The performance measures for the first set of Gaussian simulated data are presented in Table 1. Note that this table is based on 2000 simulations with 300 predictions per simulation, so 2000 total values were estimated and 600,000 points. As expected, the SLM had the lowest RMSPE, for both point and total predictions. Not only was it lowest, it was dramatically lower than any other predictor. The data were simulated with a high amount of autocorrelation, so this demonstrates how much better SLM can be in that case. When compared to MAH5 and MSN1 (the two commonly-used k-NN methods), SLM reduced RMSPE by 52.6 and 64.1% for the point estimates and 43.1 and 66.8% for the total estimates. SLM was also noticeably better than LM (linear model assuming independence), with reduced RMSPE of 34.8 and 31.8% for point and block prediction, respectively. Among the k-NN methods, MSN5 was best for both points and totals, but still not as good as LM. All methods were essentially unbiased for both points and totals. For all point estimates, prediction interval coverage was near 0.90, as they should be. For total estimates, it appears the MAH1 is a bit too high, and perhaps MAH5 a bit too low.

**Table 1:** Performance summaries from 2000 simulated spatial data sets.

	Data	P/T	MAH1	MAH5	MSN1	MSN5	bstNN	LM	SLM
RMSPE	S1	P	9.329	7.451	5.379	4.423	4.456	3.892	2.443
SRB	S1	P	−0.006	−0.009	0	−0.004	−0.004	−0.002	0.001
PIC90	S1	P	0.897	0.9	0.887	0.889	0.88	0.896	0.892
RMSPE	S1	T	262.6	289.8	174.3	153.3	154.5	139.3	87.8
SRB	S1	T	−0.058	−0.067	−0.003	−0.034	−0.034	−0.02	0.009
PIC90	S1	T	0.952	0.87	0.914	0.886	0.874	0.88	0.887
RMSPE	S2	P	6.445	5.17	6.428	5.146	5.129	5.185	4.414
SRB	S2	P	−0.024	−0.03	−0.009	−0.019	−0.030	0.000	0.003
PIC90	S2	P	0.907	0.922	0.899	0.906	0.906	0.932	0.917
RMSPE	S2	T	320	295.9	296.3	262.3	272.2	283.1	226.1
SRB	S2	T	−0.137	−0.188	−0.047	−0.135	−0.182	−0.033	−0.005
PIC90	S2	T	0.912	0.842	0.9	0.867	0.83	0.86	0.858
PCC	S3	P	0.731	0.767	0.749	0.785	0.795	0.799	0.846
SRB	S3	P	0.009	0.013	0.001	0.002	0.010	0.002	0.002
PIC90	S3	P	0.767	0.884	0.764	0.85	0.844	0.905	0.889
RMSPE	S3	T	0.0395	0.0394	0.0387	0.0334	0.0343	0.0329	0.0298
SRB	S3	T	0.072	0.09	0.003	0.019	0.079	0.018	0.014
PIC90	S3	T	0.919	0.841	0.913	0.882	0.84	0.886	0.884

In the Data column, S1 indicates data from the first simulation method, S2 indicates data from the second simulation method (count data), and S3 indicates data from the third simulation method (binary data), as described in Section “Simulation of Artificial Data.” Each data set used 100 samples per simulation, indicated by P in the P/T column, and summaries were based on 300 predictions per resampling, which were then averaged over the 2000 simulations. There was one total estimate per simulation, which were summarized over the 2000 simulations, and indicated by T in the P/T column. Different prediction methods form the rest of the columns and are described in Section “Prediction Methods.” Performance measures form the rows and are described in Section “Performance Measures;” however, note that percent correctly classified "PCC" replace RMSPE for point predictions of the binary (S3) simulated data.

The performance measures for the second set of Poisson simulated data are presented in Table 1. The SLM again had the lowest RMSPE, for both point and total prediction. SLM reduced RMSPE by 31.3 and 14.8% for point estimates when compared to MAH5 and MSN1, and SLM reduced RMSPE by 23.6 and 23.5% for total estimates. All of the methods appear to be unbiased for point prediction, with generally valid confidence interval coverage. There appears to be some bias among the k-NN methods for predicting the total, and some prediction intervals fall below 0.85 for the k-NN methods. Also, the 0.86 prediction interval coverage for the SLM was a bit low, and this simulation was its poorest performance on that measure.

For predicting a binary variable, we replaced the RMSPE with percent correctly classified (PCC) for point prediction. Only the k-NN methods with 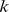 = 1 truly predicted values that were 0 or 1, so for all other methods predictions were rounded to 0 or 1. The performance measures for the binary simulated data are listed in Table 1. In fact, the k-NN with 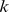 = 1 performed most poorly, with the SLM again best. SLM increased PCC by 12.5 and 10.3% over MAH5 and MSN1, respectively. Point prediction appears unbiased for all methods. Prediction interval coverage is poor for the  methods. A total of binary variables is rarely of interest in forestry applications compared to estimating proportions. For this simulation, we used the block mean, which is the estimated proportion for binary data, instead of a total. For block prediction, SLM decreased the RMSPE by 22.9 and 24.4% over MAH5 and MSN1, respectively. There may be some bias for MAH1 and bstNN. Prediction interval coverage is a little low for MAH5 and bstNN.

The performance measures for resampling real PMAI forestry data are presented in Table 2 in rows marked with PM. For point prediction, SLM reduced RMSPE by 9.0 and 34.4% over MAH5 and MSN1, respectively. Point prediction appears unbiased for all methods. Prediction interval coverage is quite good for all methods. For predicting a total, there appears to be some bias for k-NN methods using Mahalanobis distance, and prediction intervals are too large for MSN1 and too short for MAH5. The SLM reduces the RMSPE for predicting a total by 21.8 and 25.9% over MAH5 and MSN1, respectively.

**Table 2:** Performance summaries for 500 resamplings of forest data.

	Data	P/T	MAH1	MAH5	MSN1	MSN5	bstNN	LM	SLM
RMSPE	PM	P	2.998	2.371	3.243	2.53	2.362	2.399	2.127
SRB	PM	P	0.02	0.038	0.004	−0.001	0.026	0.004	0.003
PIC90	PM	P	0.895	0.902	0.888	0.894	0.898	0.897	0.899
RMSPE	PM	T	219.1	230.7	243.3	200.9	223.2	197	180.4
SRB	PM	T	0.437	0.712	0.064	−0.019	0.446	0.082	0.058
PIC90	PM	T	0.944	0.838	0.948	0.922	0.834	0.904	0.904
RMSPE	DB	P	90.8	71.3	95	73.8	68.4	69.2	67.3
SRB	DB	P	−0.002	0.000	0.000	0.002	−0.018	0.004	0.005
PIC90	DB	P	0.899	0.903	0.892	0.904	0.912	0.919	0.914
RMSPE	DB	T	6795	6369	7683	6393	6498	6193	6091
SRB	DB	T	−0.027	−0.001	0.027	0.036	−0.302	0.052	0.066
PIC90	DB	T	0.942	0.878	0.914	0.878	0.848	0.876	0.866
RMSPE	UN	P	2.983	2.497	3.115	2.495	2.389	2.436	2.146
SRB	UN	P	0.135	0.227	0.08	0.104	0.139	0.159	0.028
PIC90	UN	P	0.912	0.907	0.905	0.903	0.9	0.903	0.918
RMSPE	UN	T	637.9	853.6	457.1	442.2	576.1	608.1	269
SRB	UN	T	2.635	4.055	1.418	1.77	1.651	2.86	0.369
PIC90	UN	T	0.248	0.01	0.626	0.438	0.308	0.128	0.92

In the Data column, PM indicates the PMAI data set, DB indicates the DRYBIOT data set, and UN indicates the PMAI data set with unbalanced sampling, as described in Section “Forest Data.” Each data set used 386 samples per resampling, and for point predictions, indicated by P in the P/T column, summaries were based on 1500 predictions per resampling, which were then averaged over the 500 resamples. There was one total estimate per resample, which were summarized over the 500 resamples, and indicated by T in the P/T column. Different prediction methods form the rest of the columns and are described in Section “Prediction Methods.” Performance measures form the rows and are described in Section “Performance Measures.”

The performance measures for resampling real DRYBIOT forestry data are presented in Table 2 in rows marked with DB. For point prediction, the bestNN approached SLM for the smallest RMSPE. The MAH5 method also did quite well, but MSN1 was very poor. For predicting a total, SLM again has the lowest RMSPE. There appears to be some bias for the bestNN method. All prediction intervals are within 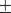 5% of 90%.

Table 2, in rows marked UN, presents the performance measures for resampling real PMAI forestry data with spatially unbalanced sampling, as shown in Figure 2. For point prediction, this creates substantially more bias than the k-NN methods in Table 2. SLM remains relatively unbiased, again with the smallest RMSPE and valid prediction intervals. For predicting a total, there are large biases for k-NN methods and prediction intervals are far from the nominal 90%. The large bias cause the RMSPE for SLM to be much lower than any k-NN methods.

**Figure 3:**
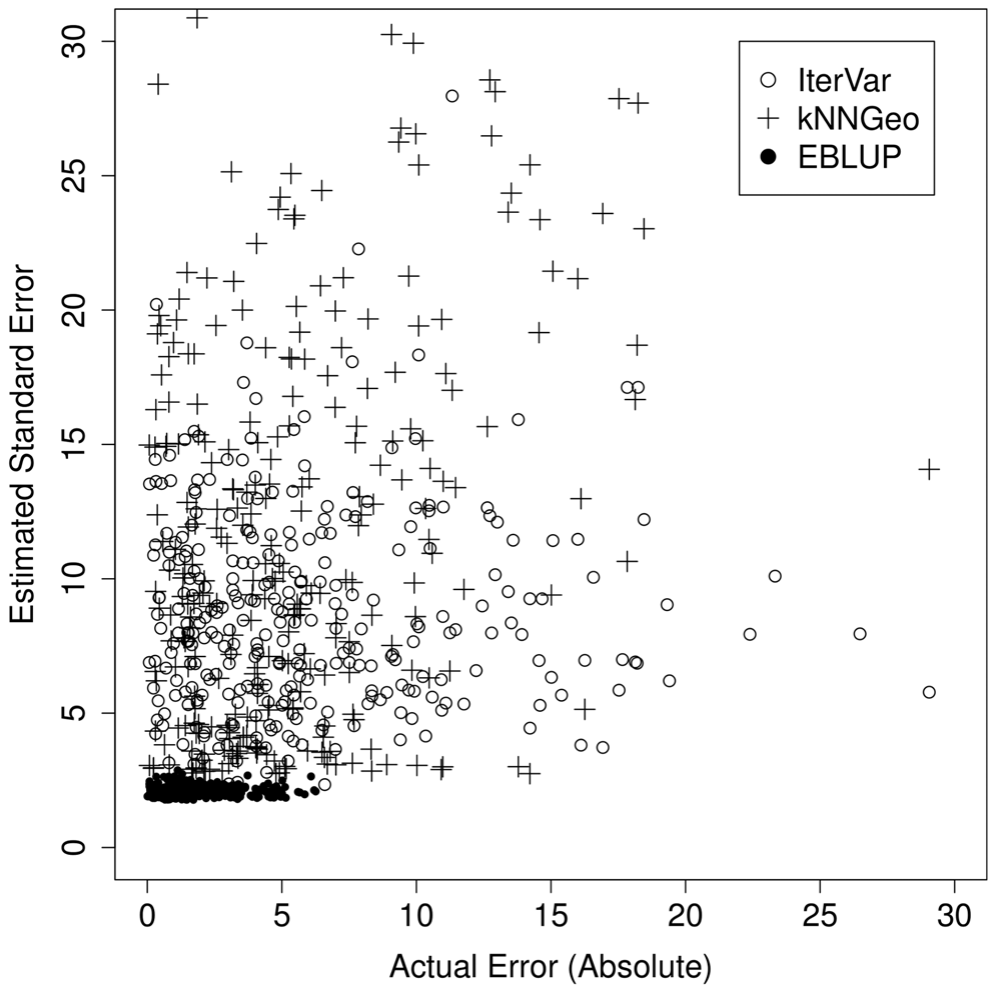
Scatter plots of absolute errors and the estimated standard errors for a single simulated data set. IterVar is the iterated variogram method of McRoberts et al. (2007), kNNGeo is the covariance matrix as estimated with all main effects in a spatial linear model and REML, but using the k-NN weights, and EBLUP are the estimated standard errors from the SLM.

**Figure 4:**
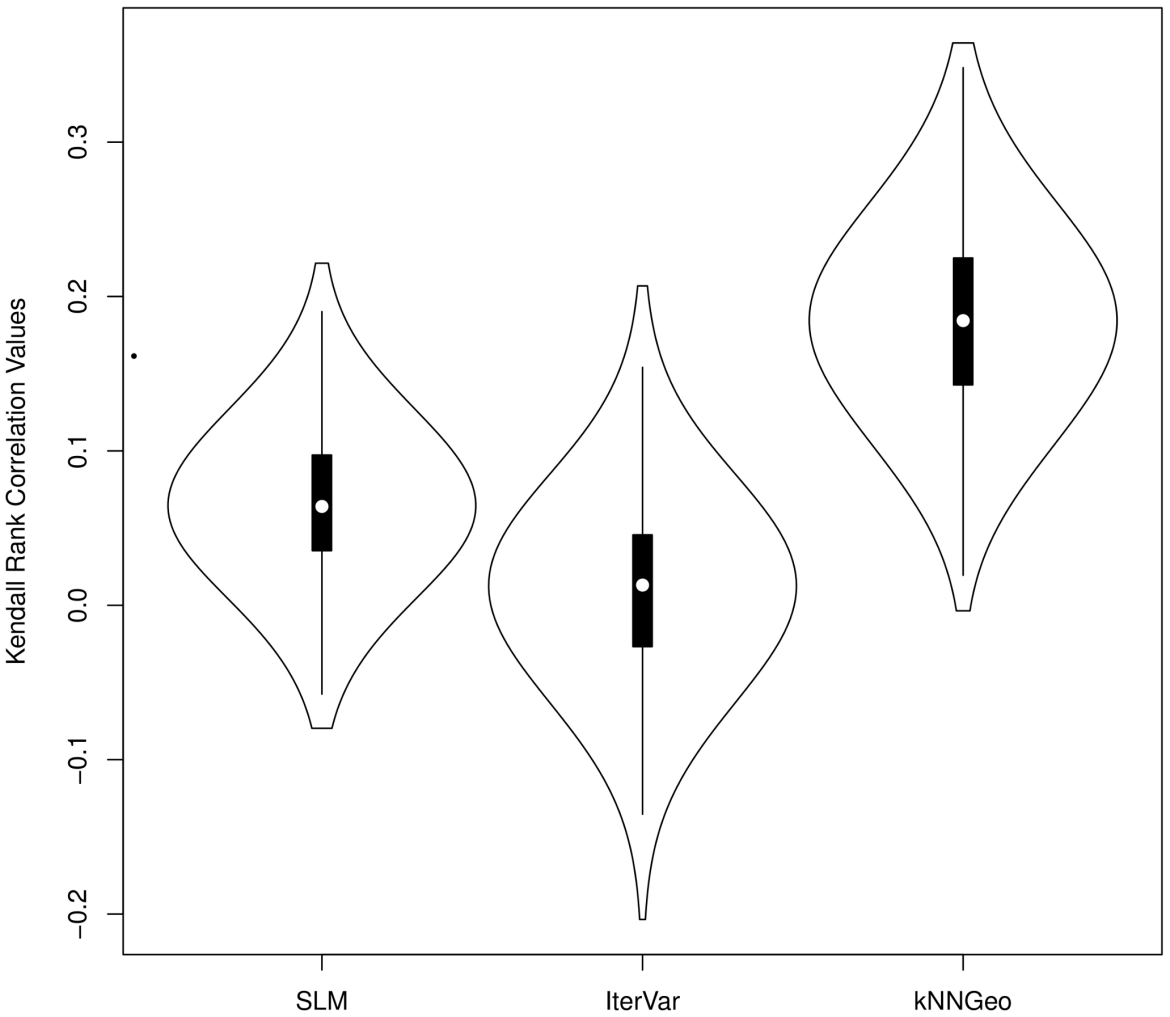
Violin plots of Kendall’s rank correlation coefficients between absolute error and estimated standard errors over 2000 simulations.

Most methods showed little bias globally, with generally valid prediction intervals. Yet, the SLM, and geostatistics in general, aims to make prediction intervals that vary in space, while the cross-validation approach used for k-NN is constant in space. We re-ran simulation 1 using the iterated variogram (IterVar) variance estimator of [23] in (18), testing its global and point-wise efficacy, compared to the SLM predictor and interval, and compared to the k-NN predictor under the SLM model (19), which we label kNNGeo. A scatter-plot of a single simulation, with 300 predictions for the unsampled locations, is shown in Figure 3, which plots 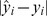 on the x-axis and 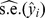 based on (9), (18), and (19) on the y-axis. We computed Kendall’s rank correlation between the true absolute error 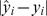 and the estimated prediction standard error, 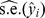 for each method. These correlations were computed for 1000 simulations, and then all correlations were plotted as violin plots for each method, which is shown in Figure 4.

Figure 4 shows that, indeed, the individual prediction intervals for the SLM are generally related to the actual errors. In contrast, Figure 4 shows that the IterVar method has no relationship between the prediction intervals and the actual absolute errors. Also, [23] claim that the algorithm is expected to rapidly converge. In our implementation, it converged only 57.4% of the time. It diverged before 30 iterations about 2% of the time. Globally, the IterVar 90% prediction interval has 88.8% coverage. The kNNGeo method of Figure 4 showed the strongest correlation between actual absolute errors and prediction intervals, largely due to the fact that it correctly estimated a dominant component of the error, which was the bias-squared. Using the RMSPE of the kNNGeo method for a 90% prediction interval had 94.2% coverage.

## Discussion

This article set out to compare k-NN to the SLM for forestry mapping, and for the estimation of totals or averages of forest resources. In the introduction, we laid out arguments that favor using k-NN, and arguments that favor using a SLM, along with disadvantages for both. Our simulations of artificial data and resamplings of real data are not exhaustive; however, for the criteria that we chose (RMSPE, signed relative bias, and prediction interval coverage), the results presented in the previous section clearly favor SLM in general. To summarize, we simulated data under conditions that should severely test the SLM method. Because k-NN is primarily used in forestry, we included various k-NN methods in the simulations. In all cases, even with mis-specified covariance models, mis-specified linear models (including nonsignificant covariates and ignoring significant ones), zero-inflated count data, binary data, and skewed real forestry data, the SLM performed better than k-NN, and generally provided valid inference with little bias, and prediction intervals that contained the true values the correct proportion of time. From a single simulation, it also appears that the SLM is more robust to unbalanced spatial sampling. These results generally verify the claim in the introduction that EBLUP used to estimate the SLM is fairly robust in a variety of ways. The SLM has an additional benefit from its model-based assumptions; it allows point-wise inference, with globally valid prediction intervals that vary at each point.

Our results can be compared to previous literature cited in the Introduction, such as [46], where our SLM is mathematically equivalent to their universal kriging (UK); however, parameter estimation likely differed in the studies ([46] do not specify if they used the REML option when they fit variograms using the GSTAT package [56]). In [46], the SLM performed well compared to another k-NN method called gradient nearest neighbor (GNN), but not as consistently better as our results. Our results can also be compared to [47], where our SLM is mathematically equivalent to their kriging-with-external-drift (KED). An interesting hybrid method that uses MSN with kriging on the residuals is compared to the SLM based on RMSPE and bias [47]. They find that the MSN-kriging hybrid is slightly better than the SLM, with both better than MSN alone. However, we note that [47] do not give a standard error estimator of point-wise predictions for the MSN-kriging hybrid. Also [47] estimate the SLM by first fitting a linear model assuming independence, and then computing and fitting a semivariogram on residuals. The use of REML for the SLM, as we have described it, estimates the fixed effects assuming correlated residuals, and is expected to be more efficient.

Note that it may seem surprising that k-NN was mostly unbiased for these simulations. Clarification is required because the Introduction claimed that k-NN is biased [16]–[19]. These authors equate bias with the fact that k-NN underestimates large values and overestimates small values; in geostatistics, this characteristic is called smoothing [51](pg. 158). Smoothing is a desirable characteristic under squared-error loss, which the SLM minimizes, so it is also a property of the SLM [57](pg. 189). Because the SLM is BLUP, it is unbiased for point-wise predictions; however the predictions are not unbiased for nonlinear functionals of the spatial population, such as quantiles. For example, following [58], but for finite populations, 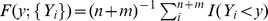 is a spatial cumulative distribution function (SCDF). Then its inverse, 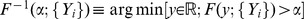 defines the 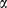 quantile, which is nonlinear in 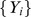. Predictors that can handle such nonlinearity have been proposed [59], and by matching variances in the predictions to those in the data, predictions will no longer underestimate high values or overestimate low values. However, it should be noted that these predictors will sacrifice the pointwise MSPE as optimized for BLUP; for an example where the prediction-variance-constrained MSPE is twice that of the “smooth” SLM predictor, see [59]. This illustrates that, in general, no set of predictors will be optimal for all purposes.

More generally, it is possible, though computationally expensive, to obtain multiple sets of predictions, where the predicted data are simulated from conditional distribution properties of the population. The multiple prediction sets can be averaged to obtain predictions that satisfy BLUP, or quantiles can be computed across the sets. Multiple sets of predictions also allow the propogation of uncertainty if prediction sets are used as inputs to other models. In fact, k-NN is closely related to multiple imputation methods [60]–[63], which sample from existing data to impute for missing data; in that sense they are like using 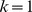 multiple times to give multiple possible “realizations.” Again, there are equivalent ideas in geostatistics, generally termed “conditional simulation,” e.g., [64] and [51](pgs. 452–453). We do not pursue a comparison here but suggest it for further research. Given the above discussion, we emphasize that our goal was point-wise unbiased prediction while minimizing the MSPE, which is what the SLM achieves in a single map, and compare that to k-NN on the same basis.

A model-based variance for k-NN predictors remains problematic. Cross-validation works from a global standpoint. Attempts at making variance local, such as the iterated variogram approach [23], did not work well for one simulated data set as shown by Figure 4. There was no correlation between estimated prediction standard errors and actual absolute errors, so cross-validation was just as good, and the iterated variogram approach had convergence problems and does not work with 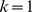. More testing of this method, and possible improvements are warranted. The kNNGeo method was correlated to actual absolute errors. However global prediction intervals for kNNGeo were too conservative with 94.2% coverage for 90% prediction intervals because the bias component is not stochastic, but is treated as such if included in prediction intervals. Thus, the SLM is the only viable choice that we examined for making valid uncertainty maps along with predictions.

Finally, we stress that the SLM was presented here as a “black box” method. As we used it, there were no decisions involved; after choosing a covariance model like the exponential, use all covariates that are available, estimate the covariance parameters with REML, and plug the resulting covariance matrix into the prediction equations. This allowed us to make predictions for thousands of simulations. Such a “black box” method is certainly possible when many predictions are needed by personnel with little statistical training. However, when data have been collected at great expense, a careful analysis is better. In that case, exploratory data analysis, understanding of covariate relationships, finding and explaining outliers, model selection and diagnostics, and finally prediction, all can enhance prediction and understanding for both the SLM and k-NN, and we recommend that over a black box approach. For example, a Bayesian approach can account for the fact that covariance parameters are estimated and should correct for the plug-in aspect of the EBLUP, e.g. [43], [65], with available software [66]. Also, when remotely sensed data are involved, data sets can be massive in size. In that case, other methods can be used, e.g. [67], [68]. Moreover, there is no single correct analysis for forestry data sets; they can be modeled in various ways to achieve different desired goals.
